# Tuning the binding behaviors of a protein YAP65WW domain on graphenic nano-sheets with boron or nitrogen atom doping†

**DOI:** 10.1039/d0na00365d

**Published:** 2020-08-26

**Authors:** Xiao Jia, Yanmei Yang, Yang Liu, Weihua Niu, Yong-Qiang Li, Mingwen Zhao, Yuguang Mu, Weifeng Li

**Affiliations:** School of Physics, State Key Laboratory of Crystal Materials, Shandong University Jinan Shandong 250100 China lwf@sdu.edu.cn; College of Chemistry, Chemical Engineering and Materials Science, Collaborative Innovation Center of Functionalized Probes for Chemical Imaging in Universities of Shandong, Key Laboratory of Molecular and Nano Probes, Ministry of Education, Shandong Normal University Jinan 250014 China yym@sdnu.edu.cn; School of Biological Sciences, Nanyang Technological University 637551 Singapore ygmu@ntu.edu.sg

## Abstract

In recent years, nanomaterials have attracted considerable research attention for biological and medical related applications due to their well-recognized physical and chemical properties. However, the deep understanding of the binding process at the protein–nanomaterial interface is essential to solve the concern of nano-toxicity. Here, we study the interactions between the recently reported graphenic nano-sheets, BC_3_ and C_3_N, and a prototypical protein (YAP65WW domain) *via* atomistic molecular dynamics simulations. Our simulations reveal that elemental doping is an effective way to tune the binding characteristics of YAP65WW with two nanomaterials. While YAP65WW can be attracted by two nanomaterials, the BC_3_ sheet is less able to disrupt the protein structure than C_3_N. From the energy point of view, this is because protein residues demonstrate a binding preference with the trend from electron rich nitrogen to electron deficient boron. Structural analyses of the bio-nano interface revealed the formation of an ordered water shell on the BC_3_ surface, which was compatible to the crystal pattern of BC_3_. When a protein binds with BC_3_, these interfacial water molecules protect the protein from being disrupted. We suggest that elemental doping is efficient to produce fruitful biological-effects of graphenic nanomaterials, which make it a prospective solution for the future design and fabrication of advanced nanomaterials with desired function.

## Abstract 2

Molecular dynamics simulations predict that atom doping is an efficient way to regulate the binding strength and structural changes of protein with nanomaterials, which makes it a prospective solution for design and fabrication of advanced nanomaterials with desired function.

## Graphical abstract

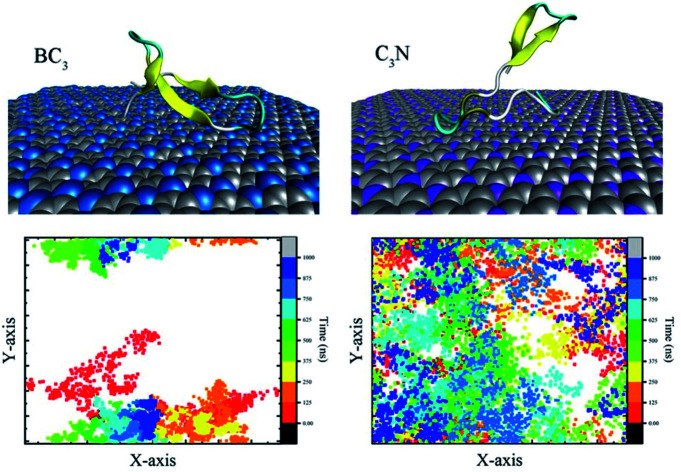

## 1. Introduction

In recent years, graphene has attracted considerable research interest in numerous fields such as electronic^1,2^ and optical materials,^3,4^ catalytic agents, sensors and biomedical materials^5^ due to its well-recognized physical and chemical properties. Particularly, in biological and medical applications, graphene has been widely studied in tumor therapy,^6^ biosensors^7^ and gene sequencing platforms.^8,9^ However, the major concern of the potential nano-toxicity has been attracting enormous attention of researchers worldwide. Numerous studies have shown that graphene can cause severe damage to biomacromolecules, such as protein unfolding, DNA unwinding, and destructive extraction of the cell membrane.^10–14^ For instance, our previous theoretical simulations demonstrate that when a graphene monolayer has defects, the protein unfolding speed is significantly accelerated by specific interactions between defects and charged residues.^15,16^ To reduce the toxicity, surface morphology engineering^17–19^ and functionalization with polyethylene glycol or serum proteins have been demonstrated to be a success.^20^ Meanwhile, our previous studies have demonstrated that a nitrogenized graphene (abbreviated as C_2_N) monolayer is biocompatible to both protein and DNA systems.^21,22^ Thus, modifying the graphenic structures by elemental doping is expected to be an efficient way to tune the biological functions of the nanomaterials.

**Fig. 1:**
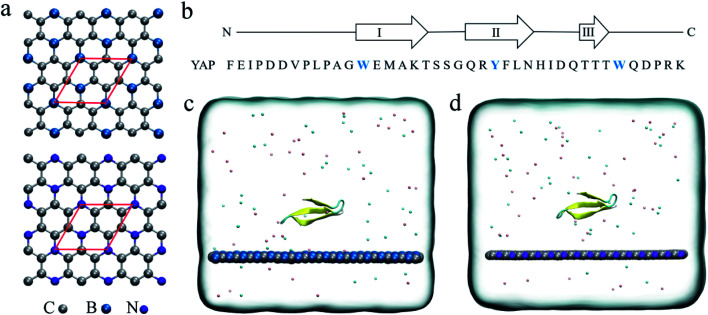
(a) Crystal structures of the BC_3_ and C_3_N monolayer. The red rhombus indicates the primitive cell of BC_3_/C_3_N. (b) The sequence of the YAP65WW domain. The positions of strands I–III, connected by hairpins, are indicated above. The initial configurations of the YAP65WW domain on the surface of BC_3_ (c) and C_3_N (d) in the simulation box. Carbon, boron and nitrogen atoms are colored in silver, blue and violet, respectively. Na^+^ and Cl^−^ ions are shown as pink and cyan spheres. Water molecules are not shown for clarity.

Recently, the successful experimental syntheses of boron-doped graphene, BC_3_ (ref. 23) and nitrogen-doped graphene, C_3_N^24^ have been achieved. BC_3_ and C_3_N monolayers have excellent material chemistry properties and are used in batteries^25–27^ and high sensitive gas sensors.^28–32^ Structurally, the B and N atoms are arranged in a patterned manner in graphenic nanosheets. Each B or N atoms is covalently connected to three C atoms (Fig. 1a). Due to the electronegativity difference between the three constitute elements, B, C and N, there is an intrinsic electron transfer within the planar structure of BC_3_ and C_3_N, resulting in periodically distributed dipoles. A recent theoretical study on the interactions of BC_3_ and water clearly showed that BC_3_ induced a significant re-arrangement of water dipoles at the interface, making BC_3_ a promising candidate for the transport of drug species.^33^ Thus, element doping to graphene is expected to bring out interesting behaviors at the nano-bio interface compared to the non-polar graphene.

In this study, all-atom molecular dynamics (MD) simulations have been conducted to explore the dynamic and energetic characteristics of a prototypical protein, YAP65WW domain, binding with BC_3_ and C_3_N nanosheets. The adsorption processes and structure evolutions of the protein were systematically analyzed and compared. The simulation results revealed that both BC_3_ and C_3_N were capable of attacking YAP65WW. It is interesting to find that C_3_N is more capable of inducing the structural unfolding of YAP65WW. This mechanism is because of the binding preference of the protein with the trend from electron-rich nitrogen to electron-deficient boron. We believe that the perspective insights obtained from our study would be beneficial to guide and potentiate the practical applications of graphenic materials and bring about a flourishing new branch in the design of functional bio-nano materials.

## 2. Computational methods

The initial coordinates of YAP65WW were obtained from the crystal structure (PDB code 1JMQ^34^ and truncated to include residues 15–40 (ref. 16)). We selected YAP65WW as a model protein because it has been widely used in the study of protein folding kinetics and the interactions with nanomaterials.^35–42^ As shown in Fig. 1b, YAP65WW is a signaling and regulatory protein, which exists as a three-stranded, antiparallel β-sheet topology.^43–46^ The BC_3_ nano-sheet has dimensions of 7.76 × 8.06 nm^2^ and is composed of 1620 carbon and 540 boron atoms. Similarly, C_3_N contains 1620 carbon and 540 nitrogen atoms with dimensions of 7.29 × 7.59 nm^2^. The nano-sheet was placed in the *xy* plane and the cross-section of the simulation box was equal to the size of the nano-sheet. Following our previous study,^21^ the positions of C_3_N and BC_3_ nano-sheets were restrained using a harmonic potential with a force constant of 1000 kJ mol^−1^ nm^−2^ in all the simulations to mimic a local surface in the whole sample. As depicted in Fig. 1c and d, YAP65WW was then placed above the nano-sheet with a separation of 2 nm (measured from the center of mass). Then, the complex was solvated in a water box with a height of 7 nm. Periodic boundary conditions were applied on the three directions. For water, the SPC/E water model^47,48^ was used. Then, 40 Na^+^ and 42 Cl^−^ ions were added to neutralize the net charge of YAP65WW and mimic the physiological condition.

The MD simulations were performed using the GROMACS package.^49^ The YAP65WW was described by the AMBER99sb force field.^50^ For the two nano-sheets, the force field parameters were adopted from our previous work.^18^ The long-range electrostatic interactions were treated using the particle mesh Ewald (PME) method.^51,52^ The van der Waals (vdW) interactions were calculated with a cutoff distance of 1.2 nm. The covalent bonds involving hydrogen atoms were constrained by the LINCS algorithm.^53^ After energy minimization, both systems were equilibrated for 1 ns in the NVT ensemble using the v-rescale thermostat at 300 K,^54^ and simulated for another 1 ns in the NPT ensemble with a pressure coupling of 1 bar at the *z*-direction of the simulation box and temperature of 300 K using the Parrinello–Rahman coupling method. During these two stages, position restrains were applied to the heavy atoms of the protein. Then, 1000 ns production simulations were conducted in the NVT ensemble at 300 K. For each nano-sheet, three parallel simulations with different protein orientations and initial velocities were generated for data analysis. In addition to the BC_3_ and C_3_N simulations, three control simulations of YAP65WW in water solvent have also been conducted with similar setups to verify the intrinsic stability of protein itself.

## 3. Results and discussions

### 3.1 Quick loading of YAP65WW onto BC_3_ and C_3_N

**Fig. 2:**
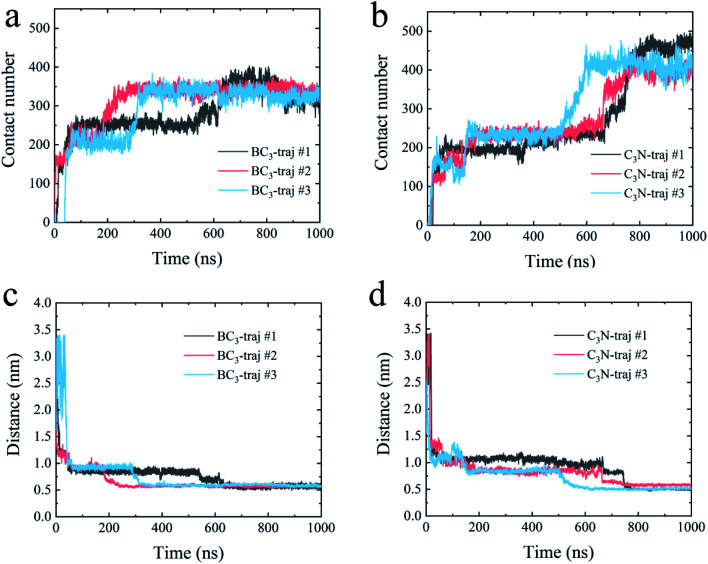
The contact number between the YAP65WW and BC_3_ (a) and C_3_N (b); the separation distance between the center of mass of YAP65WW and BC_3_ (c) and C_3_N (d) along the normal directions of the nanosheets.

First, we accessed the binding behavior of YAP65WW with two substrates. Initially, YAP65WW was placed above each substrate with a distance of 2 nm. As summarized in Fig. 2, the separation dropped to around 1.2 nm during the first tens of nanoseconds in the simulations (stage one), indicating the quick loading of the protein to the substrates. Then, the separations uniformly decreased to around 0.55 to 0.60 nm in three trajectories after hundred nanoseconds of simulations (stage two), although the specific time differed in different trajectories. A similar tendency was also observed when monitoring the number of contacts (*N*_c_) between the protein and the substrates. Along with the decrease in distance, YAP65WW established firm contacts with the two substrates. A pair of atoms from the protein and BC_3_/C_3_N, respectively, separated within 0.6 nm was defined as a contacting pair. The *N*_c_ profiles first quickly increased to a plateau of around 80 within 100 ns in two simulations, except that it took a slightly longer time (∼150 ns) to reach this plateau in the third trajectory for C_3_N, corresponding to the first stage in the distance analyses. Then, in the second stage, YAP65WW adjusted its conformation until the end of the simulations. It is worth noticing that YAP65WW demonstrated different adsorption behaviors on BC_3_ and C_3_N at the end of the simulations. In detail, there are 124.38 ± 2.64 and 157.90 ± 4.61 atomic contacting pairs (each value is averaged over three trajectories) for binding of YAP65WW to BC_3_ and C_3_N, respectively, which accounted for 57.58 ± 1.22% and 73.10 ± 2.13% of heavy atoms of the protein. From this phenomenon, it is expected that C_3_N is energetically more attractive to YAP65WW because more intimate bindings are established.

### 3.2 Structure disruption of YAP65WW and key residues at the interface

**Fig. 3:**
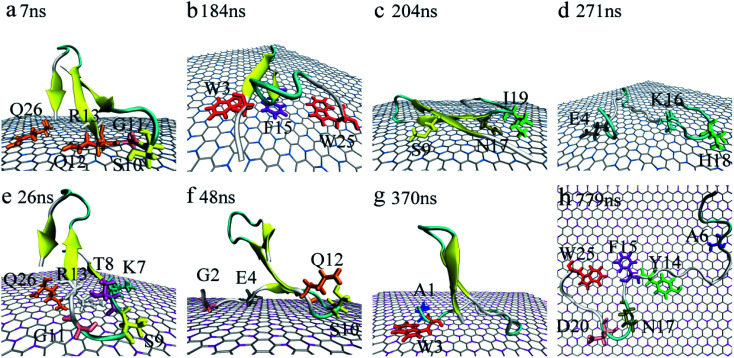
Structure evolution of the YAP65WW domain during the adsorption process on the surface of BC_3_ (a–d) and C_3_N (e–h). Residues that play key roles at the bio-nano interface are highlighted.

Accompanied by the binding, obvious structure changes were observed for YAP65WW on two nano-sheets. Fig. 3 depicts eight representative snapshots for YAP65WW binding with BC_3_ and C_3_N. At the early binding stage, YAP65WW approaches to the BC_3_ atoms at ∼7 ns (Fig. 3a) through five residues, S10, G11, Q12, R13 and Q26 (the contacting time is determined by monitoring the *N*_c_ of each residue with BC_3_/C_3_N, as depicted in Fig. S1 in the ESI†). Then, the protein laid down with its β-sheets stacking parallel to the BC_3_ surface (Fig. 3b). Three aromatic key residues, W3, F15 and W25, were found to form intimate π–π stacking^55^ with BC_3_, which caused a quick flipping over for the strand-3 and more residues (S9, N17 and I19) adsorbed on the BC_3_ surface (Fig. 3c). Then, the protein was found to undergo a severe unfolding process (Fig. 3d).

While for C_3_N, YAP65WW contacted to C_3_N at 26 ns by the K7, T8, S9, G11, R13 at the β-hairpin region and Q26 (Fig. 3e). At about 48 ns, the strand-1 of YAP65WW formed firm contacts with C_3_N through G2, E4, S10 and Q12 (Fig. 3f). The unfolding of the β-sheets happened at ∼370 ns at strand-1 (Fig. 3g). At around 749 ns, YAP65WW completely unfolded (Fig. 3h) with most residues (A6, Y14, F15, N17, D20 and W25) adsorbed on the C_3_N surface. Despite the different binding processes, it is notable that the π–π stacking interactions between the aromatic residues and the substrate appear in both systems. As shown in Fig. S2,† the interfacial water molecules were squeezed once the aromatic residues approached, resulting in nano-scale dewetting. From previous studies, the π–π stacking is commonly found in the interactions of graphenic nanomaterials with aromatic groups of biomolecules, resulting in severe structural distorsions.^15,16,55–58^

**Fig. 4:**
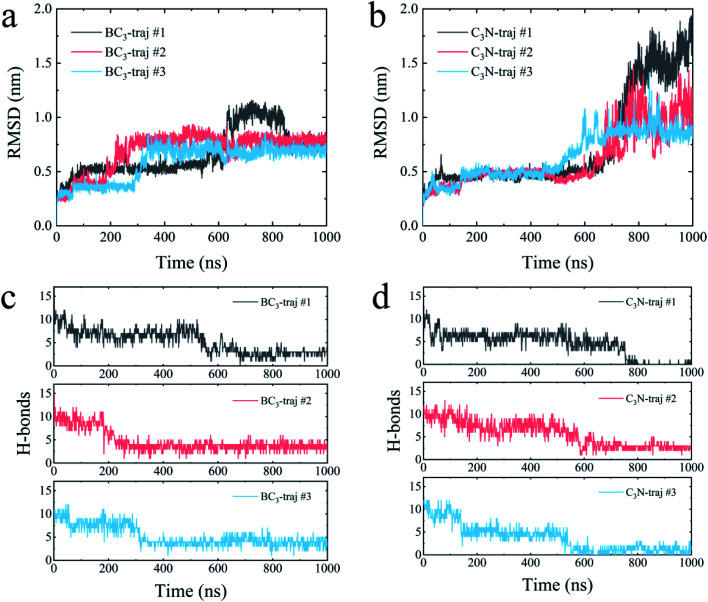
Time evolutions of the root mean square deviation (RMSD) of the YAP65WW heavy atom relative to the crystal structure upon binding to BC_3_ (a) and C_3_N (b). Time evolution of number of intra-protein H-bonds upon binding to BC_3_ (c) and C_3_N (d).

We further quantitatively accessed the structural evolution of the protein by calculating the root mean squared deviation (RMSD) of YAP65WW with respect to the crystal structure (only heavy atoms were used in the calculations). As summarized in Fig. 4, the RMSD profiles first increased to around 0.5 nm, particularly for YAP65WW with C_3_N, revealing a slight conformational change from the native structure at the early binding stage. For YAP65WW binding to BC_3_, the totally unfolded state has an RMSD value of around 0.75 nm (Fig. 4a). In contrast, for the case of YAP65WW on C_3_N, RMSD reached 0.8, 1.0 and 1.75 in three trajectories, indicating that the protein structure change is more severe (Fig. 4b). In the control simulations of an isolate YAP65WW in water, the protein maintained a highly ordered native structure with no obvious unfolding being observed (Fig. S3†). This confirms that the unfolding of YAP65WW in the BC_3_ and C_3_N simulations is mainly caused by the nano-surface.

It is well known that the spatial structure of protein is mainly maintained by the intra-protein hydrogen bonds (H-bonds). During the unfolding process, a clear decrease in the number of H-bonds was observed. As shown in Fig. 4c, the H-bonds decreased from 17 for native YAP65WW to 2.89 ± 0.56, 3.57 ± 0.78 and 3.77 ± 0.99 after 1000 ns simulations for YAP65WW binding to BC_3_. However, for C_3_N (Fig. 4d), relatively less H-bonds were maintained and the corresponding values were 0.10 ± 0.30, 2.71 ± 0.56 and 0.95 ± 0.83. This phenomenon confirms that C_3_N causes more severe YAP65WW disruptions than BC_3_. It is also worth mentioning that, although BC_3_ induced a smaller ratio of structure loss in YAP65WW, the beginning of YAP65WW unfolding on the BC_3_ surface was earlier than that on C_3_N.

### 3.3 Secondary structure evolution of YAP65WW

**Fig. 5:**
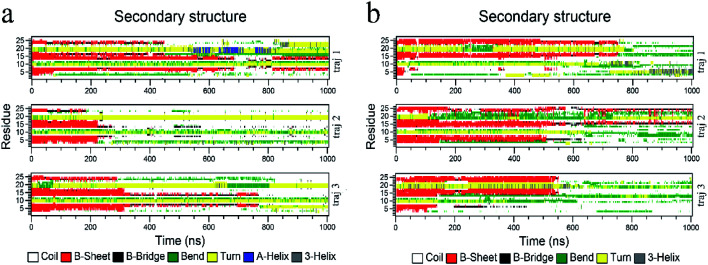
Time evolution of the secondary structure of YAP65WW upon binding to BC_3_ (a) and C_3_N (b). The calculations of the secondary structures were conducted by the do_dssp module implemented in the GROMACS package.

The biological functions of proteins mainly depend on the secondary structure. Considering this, the in-depth secondary structure analyses of YAP65WW were further conducted using the define secondary structure of protein (DSSP)^59^ program, as summarized in Fig. 5. At the native conformation, YAP65WW contains three antiparallel β-sheet segments: β-sheet 1 (residues 2–8), β-sheet 2 (residues 12–18), and β-sheet 3 (residues 21–25). These three segments were initially preserved during the first 200–300 nanosecond simulations, after which the β-sheets almost disappeared except that small segments showed a dynamic manner of unfolding and folding in the first trajectory of the YAP65WW–BC_3_ system and the second trajectory of the YAP65WW–C_3_N system. At the end of all the trajectories, no obvious segments with ordered secondary structures were maintained.

### 3.4 Transverse diffusion of YAP65WW on BC_3_ and C_3_N

**Fig. 6:**
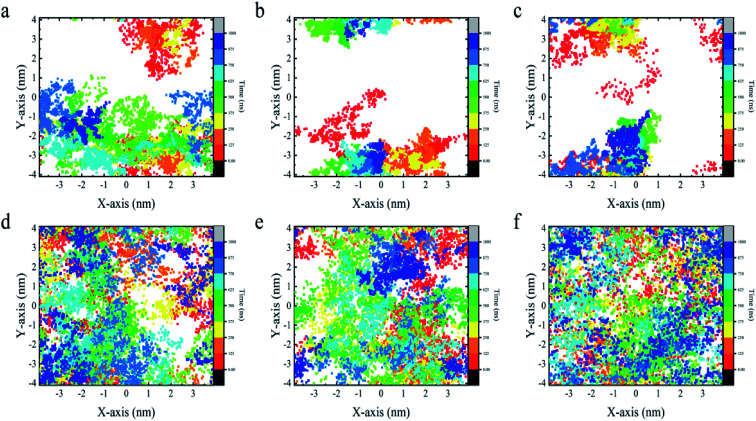
Transverse diffusion of YAP65WW on the surface of BC_3_ (a–c) and C_3_N (d–f) from three parallel trajectories for each substrate. Each dot in the figure depicts the position of the center of mass of protein at certain time.

From the above analyses, both BC_3_ and C_3_N are found to be rather attractive to protein because the YAP65WW binding was found to be constantly preserved throughout the simulations. However, differences were also observed between the two substrates. In Fig. 6, we plotted the transverse diffusion of YAP65WW on the two surfaces, which revealed distinct behaviors. For BC_3_, the diffusion of protein is relatively slow, as shown in Fig. 6a–c. During the 1000 ns simulations, the protein only travelled to certain regions of the BC_3_ surface. In contrast, during the 1000 ns simulations, the trajectories of YAP65WW almost covered the C_3_N surface, as shown in Fig. 6d–f. This reveals that the transverse diffusion of protein on the BC_3_ surface is, to a certain extent, restricted. To further understand the reasons for the different diffusivities, we quantitatively analyzed the bio-nano interface. In Fig. S4 in the ESI,† we plotted the distributions of surface water on BC_3_ and C_3_N, respectively. It was found that water molecules on BC_3_ form a highly ordered structure, which is compatible with the crystal pattern of BC_3_. On the top of hexagonal carbon rings, obvious water clusters are found with high densities. When protein binds with BC_3_, these localized water clusters will definitely hinder the protein diffusion and protect protein from being denatured.

### 3.5 Energetic characteristics of YAP65WW binding to BC_3_ and C_3_N

**Fig. 7:**
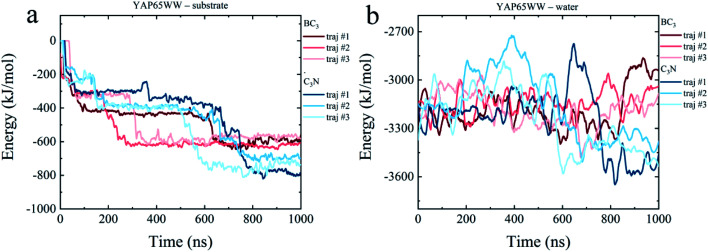
Time evolutions of interaction energy terms of (a) YAP65WW – substrate and (b) YAP65WW – water.

To dissect the origins of the driving force for the YAP65WW and BC_3_/C_3_N binding, we have analyzed the interaction energy components from: (i) YAP65WW interacts with substrate and (ii) YAP65WW interacts with water. As shown in Fig. 7a, the adsorptions of YAP65WW to BC_3_ and C_3_N are both exergonic. The YAP65WW–C_3_N binding releases more energy (averagely 752.20 kJ mol^−1^) than the YAP65WW–BC_3_ (averagely 589.15 kJ mol^−1^), indicating more intimate binding of the former. Particularly, for YAP65WW–C_3_N from 500–800 ns, sharp decreases can be observed for the energy profiles that are consistent with the *N*_c_ analyses in Fig. 2a.

The energy term representing the direct interactions of YAP65WW and substrate drives the binding process. In general, the energy released from binding partially compensates the increase in intra-protein potential energy that maintains the folded state. In addition, the interaction energy of the protein with water molecules (abbreviated as *E*_hydra_) also changes because of the conformational unfolding upon adsorption. As summarized in Fig. 7b, for the two systems, the time evolutions of two *E*_hydra_ are found to be quite different. Particularly, it is interesting to find a clear energy decrease for YAP65WW – water in the C_3_N system at 400–700 ns. We attribute this phenomenon to the more severe unfolding of YAP65WW on C_3_N than that on BC_3_, as revealed by the RMSD in Fig. 4a and b. This resulted in more residues exposed to water, which lower the interaction energy to water solvent. This is further supported by monitoring the contacting water around YAP65WW.

**Fig. 8:**
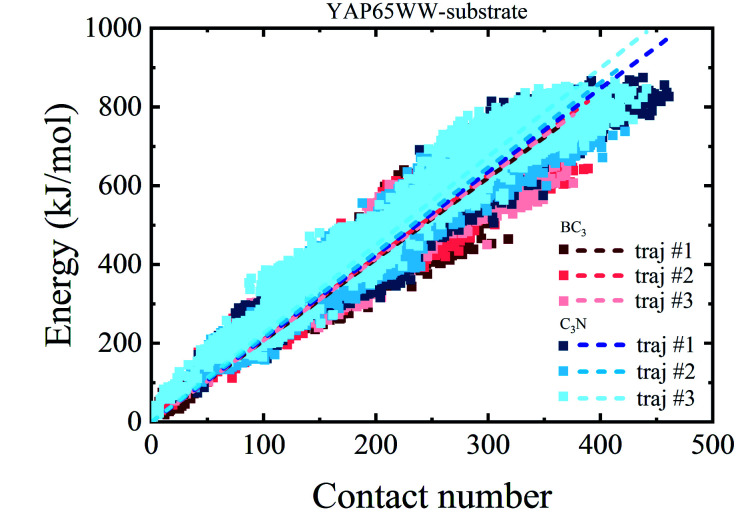
Relationship between the interaction energy and number of contacts between YAP65WW and substrate (the dash lines indicate linear fit of all data from whole trajectories).

To provide a deep understanding of the atomic contribution to the overall binding energy, we plotted the dependence of interaction energy between YAP65WW and substrate with respect to number of contacts (*N*_c_) between them (only heavy atoms are considered). As summarized in Fig. 8, a clear linear dependence of the binding strength on *N*_c_ was observed. Second, the larger slopes of the C_3_N–protein represent a stronger binding strength than BC_3_–protein. This reflects that C_3_N is more attractive towards protein, which is consistent to Fig. 7a. Further decomposition of total interaction energy to the elemental level (Fig. S5†) indicates a binding preference with the trend from nitrogen boron. Based on these observations, it is believed that elemental doping can efficiently regulate the interactions of graphenic materials with biomacromolecules, which deserve further experimental efforts.

## 4. Conclusion

Using MD simulations, the characteristics of the binding process and structural evolution of protein with graphenic layered materials, B_3_C and C_3_N, were systematically studied. The effects of the elemental doping of boron and nitrogen atoms were compared. Our data clearly revealed that both BC_3_ and C_3_N are rather attractive to protein. The binding further induced distortions in protein secondary structures. More importantly, C_3_N demonstrated a stronger capability to destroy the protein structure than BC_3_. In terms of interaction energy, the binding preference of protein follows the trend from electron-rich nitrogen to electron-deficient boron. Localized high density water clusters are formed on the B_3_C surface, which can effectively hinder the transverse diffusion of the protein on it. These findings could be useful to guide the design and fabrication of novel layered nanomaterials with fruitful and tunable biological effects through the elemental doping technique.

## Conflicts of interest

There are no conflicts to declare.

## Supplementary Material

NA-002-D0NA00365D-s001
