# Supplementary material for: Tuning the binding behaviors of a protein YAP65WW domain on graphenic nano-sheets with boron or nitrogen atom doping
Source: Nanoscale Adv. 2020 Aug 26;2(10):4539–46. doi: 10.1039/d0na00365d (PMC9417744; doi:10.1039/d0na00365d)
Supplement: NA-002-D0NA00365D-s001 [file NA-002-D0NA00365D-s001.pdf]

Electronic Supplementary Material (ESI) for Nanoscale Advances.
This journal is © The Royal Society of Chemistry 2020

Supplementary Information

Tuning the Binding Behaviors of Protein YAP65WW Domain on Graphenic Nano-Sheets with Boron or Nitrogen Atom Doping

Xiao Jia[1], Yanmei Yang[2]*, Yang Liu[1], Weihua Niu[2], Yong-Qiang Li[1], Mingwen Zhao[1] Yuguang Mu[3]* and Weifeng Li[1]*

1. *School of Physics and State Key Laboratory of Crystal Materials, Shandong University, Jinan, Shandong, 250100, China*
2. *College of Chemistry, Chemical Engineering and Materials Science, Collaborative Innovation Center of Functionalized Probes for Chemical Imaging in Universities of Shandong, Key Laboratory of Molecular and Nano Probes, Ministry of Education, Shandong Normal University, Jinan, 250014, China*
3. *School of Biological Sciences, Nanyang Technological University, 637551, Singapore*

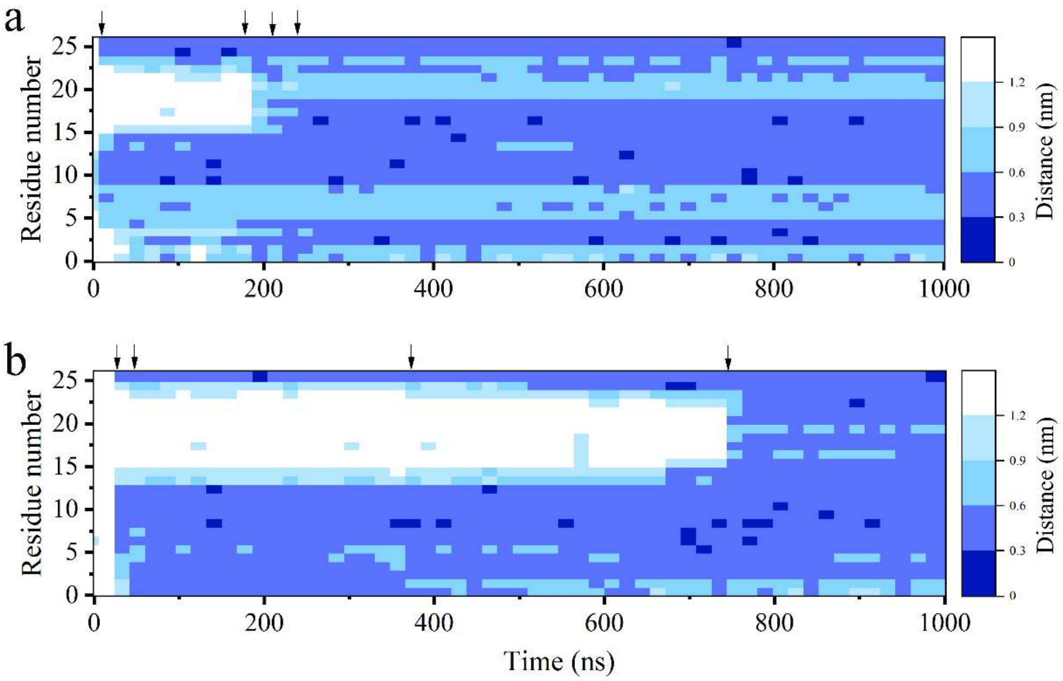

Figure S1. The evolutions of the contact state of protein residues with (a) BC_3 and (b) C_3N. Deep blue indicates formation of intimate contacts.

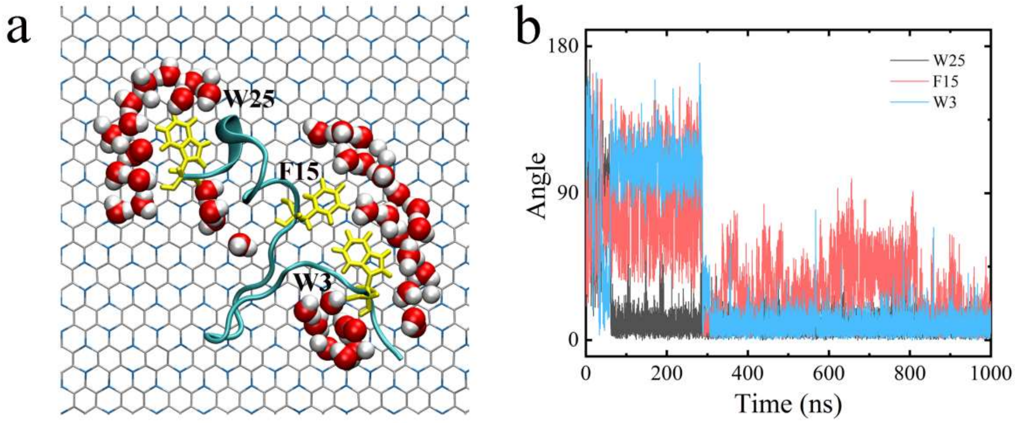

Figure S2. (a) The top view of YAP65WW on BC_3 at 1000ns. The water molecules within 0.6nm to both BC_3 and contacting residues (W3, F15 and W25) are shown as spheres. (b) Time evolutions of the stacking angle between contacting aromatic residues (W3, F15 and W25) and the BC_3 sheet. Angles are defined by the normal directions of the aromatic rings and the z-axis (normal direction of BC_3 sheet). Thus, a value of 0 degree represents face-to-face stacking.

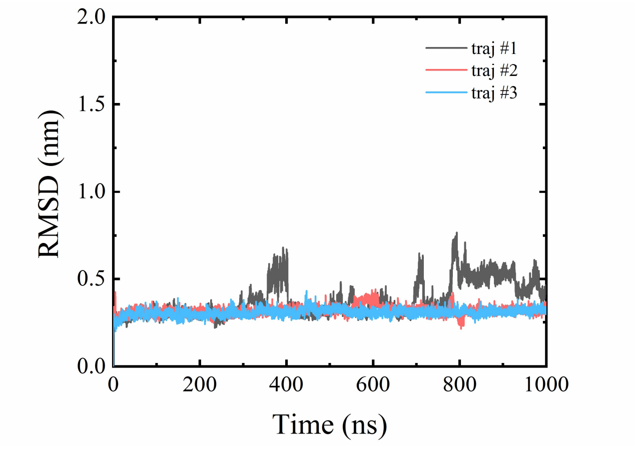

Figure S3. Time evolutions of the root-mean-squared deviation (RMSD) of the YAP65WW heavy atoms in water with respect to the crystal structure in three trajectories.

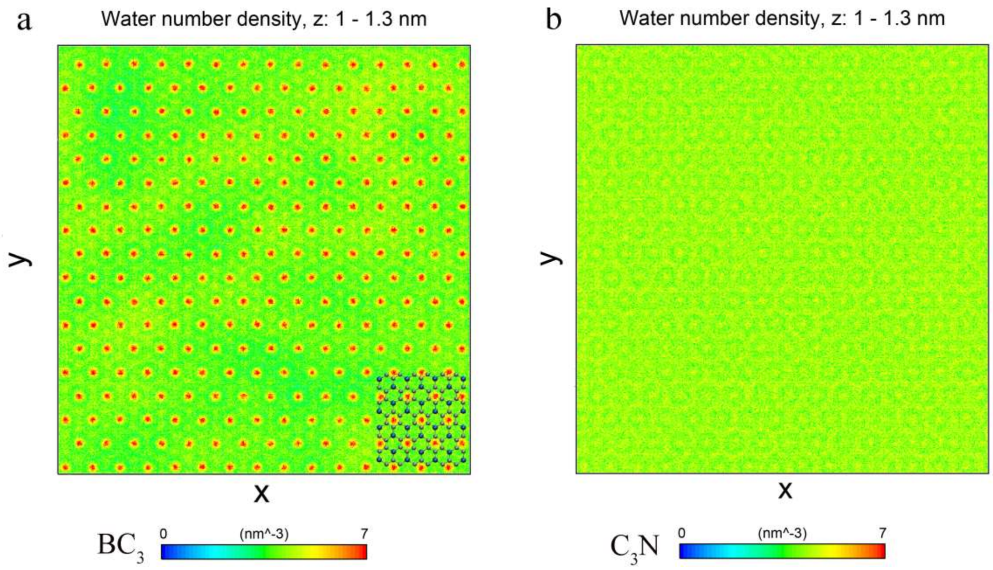

Figure S4. Two dimensional density contour map of the solvation water shell of (a) BC_3 and (b) C_3N.

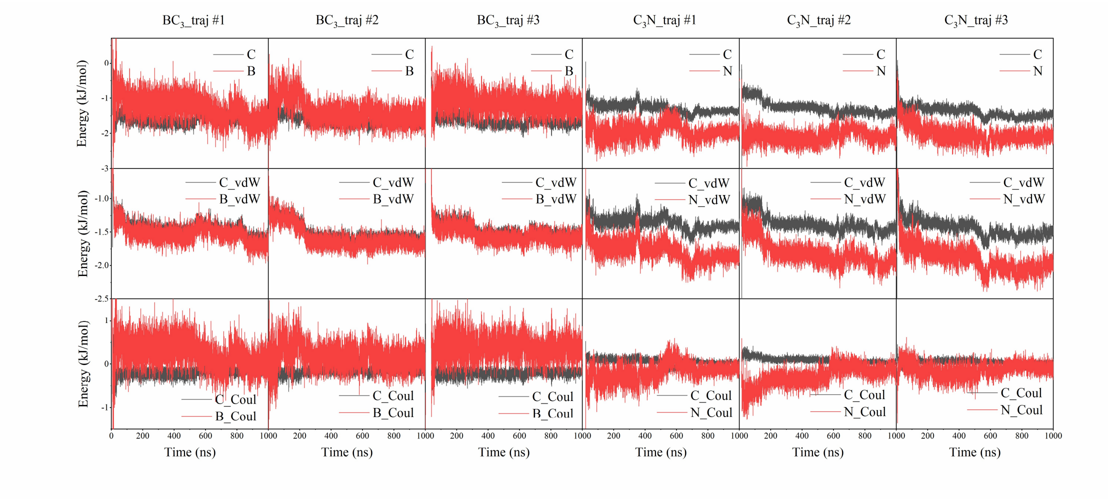

Figure S5. The time evolutions of total interaction energy, van der Waals (vdW) and Coulomb (Coul) contributions between protein and atoms in BC_3/C_3N.
